# Supplementary material for: Development, validation, and pilot MRI safety study of a high-resolution, open source, whole body pediatric numerical simulation model
Source: PLoS One. 2021 Jan 13;16(1):e0241682. doi: 10.1371/journal.pone.0241682 (PMC7806143; doi:10.1371/journal.pone.0241682)
Supplement: S1 File — (DOCX) [file pone.0241682.s001.docx]

**Supporting Information:**

**Development, Validation, and Pilot MRI Safety Study of a High-Resolution, Open Source, Whole Body Pediatric Numerical Simulation Model**

Hongbae Jeong^1,2^, Georgios Ntolkeras^1,3^, Michel Alhilani^3,4^, Seyed Reza Atefi^1,2^, Lilla Zöllei^1,2^, Kyoko Fujimoto^5^, Ali Pourvaziri^2^, Michael H. Lev^2^, P. Ellen Grant^3^, Giorgio Bonmassar^1,2*^

*^1^ Athinoula A. Martinos Center for Biomedical Imaging, Massachusetts General Hospital, Harvard Medical School, Boston, MA, USA.*

*^2^ Department of Radiology, Massachusetts General Hospital, Harvard Medical School, Boston, MA, USA.*

*^3^ Fetal-Neonatal Neuroimaging and Developmental Science Center, Boston Children’s Hospital, Harvard Medical School, Boston, MA, USA.*

*^4^ Department of Medicine, Charing Cross Hospital, Imperial College Healthcare NHS Trust, London, United Kingdom*

*^5^ Center for Devices and Radiological Health, U.S. Food and Drug Administrator, Silver Spring, MD, United States*

Contents

[S1 Appendix. 2](#_Toc52449786)

[S2 Appendix 4](#_Toc52449787)

# S1 Appendix.

Contains additional information on the i) SAR and Skin-depth calculation, ii) a detailed list of tissues in the model generated by automated segmentation and manual segmentation, Tissue property assignment for the tissues for which no measurements have been published, Uncertainty Analysis, and the hand bone age validation.

***‘Appendix***

Specific Absorption Rate was calculated using the following equation,

The SAR (W/kg) is calculated at every location in the model, that is

$$SAR\left( r \right)=\frac{\sigma(r)}{2\rho(r)}\left| E(r) \right|^{2}$$

(1)

where $\sigma(r)$ is the local tissue conductivity at location r, $\rho(r)$ is the local mass density at location r, $\left| E(r) \right|$ is the electric field at location r, and r is the location of the model

The SAR can be averaged over a specified volume or mass. The SAR_head_ is given by averaging over the volume of the head,

$${SAR}_{Head}=\frac{1}{V_{Head}}\int_{V_{Head}} \frac{\sigma\left( r \right)}{2\rho\left( r \right)}\left| E\left( r \right) \right|^{2}dV$$

(2)

Whole-body averaged SAR (SAR_wb_) is found in the same fashion in the volume of whole-body volume.

Similarly, 10gSAR is calculated by averaging any ten-grams of tissue in the shape of a cube volume,

$$10gSAR \left( r \right)=\frac{1}{V_{10g}}\int_{V_{10g}} \frac{\sigma\left( r \right)}{2\rho\left( r \right)}\left| E\left( r \right) \right|^{2}dV$$

(3)

Skin depth calculation of 29-month-old child in 1.5 T

Skin depth, $\delta,$is the RF penetration inside a conductive sample, that is given by

$$\delta=\sqrt{\frac{2}{\omega\mu\sigma}}$$

(4)

where $\omega$ is the angular frequency, $\mu$ is the magnetic permeability of vacuum ($4\pi\times{10}^{-7}$H/m), $\sigma$ is the tissue conductivity (1).

Incident tangential E-field calculation on AIMD in 1.5 T for using in Tier-3 analysis

Examples of simulations followed the newest (2018) International Organization for Standardization Technical Specification (ISO/TS) 10974 Technical Specification – assessment of the safety of magnetic resonance imaging for patients with an active implantable medical device (2). Sim4Life (3) (ZMT, Switzerland) was used to solve the Maxwell equation at 64 MHz. 16-rung high-pass birdcage coil (diameter: 290 mm, length: 290 mm) tuned to 64 MHz (S_11_ < -14 dB) and was used to generate a B_1_ transmit field with circularly polarized mode with an RF shield (4) (**Fig 11**). The results of EM fields (without a VNS implant) were normalized to 3.2 W/kg (maximum allowed RF exposure in head MRI scanning (5)) and used for the calculation of incident tangential E-field (E_tan_) on clinically potential lead trajectories using IMSAFE tool in Sim4Life. The baseline trajectory was chosen by the neurologist’s guidance, and 20 random paths were generated within the disc radius of 2 mm along the VNS lead trajectory with the constraint not to travel on bones, skin, background, lungs, and heart. The results of the incident E_tan_ on our MARTIN model are shown in **S1 Fig** as an example of EM simulation use in Tier-3 analysis followed by ISO/TS 10974: 2018 (2).

# S2 Appendix

Contains additional information on the existing pediatric numerical models.

‘Appendix

Various children models are introduced or morphed from an older age group, such as the Nina from the Virtual Family (6), the GSF family (Baby, Child) by Helmholtz Zentrum München (7), the pediatric xCAT phantom (8), and Chinese family (9). The Baby model in the GSF family is also used for the development of the Charlie model for Sim4Life users. However, these models have gaps in the validation of their segmentation anatomical details as the anatomy may contain inaccuracies due to the anisotropic growth of the different tissues, as shown in **Table 1**. These models are designed and used for neonatal numerical modeling (10), but not represent 29-month-old well. For example, a Baby model in the GSF family has an incomplete tissue segmentation of the brain (7), which is essential for implant RF safety assessment in MRI. A model with detailed structures will allow for the correct anatomical placement of implants and study the potential consequences or side effects of heating in the surrounding tissues. Furthermore, the currently available children numerical models younger than five have been morphed from older body models (7,8,11,12), which may produce anatomical inaccuracies (13) since the body tissues do not grow proportionally during childhood.

**S1 Table**. **Tissues segmented automatically using an automated segmentation tool** (14). See **Fig 4** for the results of the automated segmentation.

| Count | Brain Tissues |
| --- | --- |
| 1 | L/R Cerebral White Matter |
| 2 | L/R Cerebral Cortex |
| 3 | L/R Lateral Ventricle |
| 4 | L/R Cerebellum White Matter |
| 5 | L/R Cerebellum Cortex |
| 6 | L/R Thalamus |
| 7 | L/R Caudate |
| 8 | L/R Putamen |
| 9 | L/R Pallidum |
| 10 | 3rd-Ventricle |
| 11 | 4th-Ventricle |
| 12 | L/R Hippocampus |
| 13 | L/R Amygdala |
| 14 | L/R Accumbens area |
| 15 | L/R Ventral DC |
| 16 | Vermis |
| 17 | Midbrain |
| 18 | Pons |
| 19 | Medulla |

**S2 Table***.* **List of the main bones that were segmented.**

| Skull | Scapulae | Sacrum | Tibia |
| --- | --- | --- | --- |
| Upper and lower mandible | Humerus | coccyx | Fibula |
| Vertebrae (cervical, thoracic, lumbar) | Radius | Ilium | Tarsal bones |
| Scapulae | Ulna | Ischium | Metatarsal bones |
| Clavicles | Carpal bones | Pubic bone | Phalangeal bones Foot |
| Sternum | Metacarpal bones | Pelvic bones |  |
| Ribcage | Phalangeal bones hand | Femur |  |

**S3 Table**. **Main muscular compartments were segmented.**

| L/R anterior thigh muscles | L/R temporalis | Muscles of the back | Muscles of the head and face |
| --- | --- | --- | --- |
| L/R posterior thigh muscles | L/R Gluteus | Muscles of the forearm | Muscles of the neck |
| L/R biceps | L/R Iliopsoas | Muscles of the arm | Periocular muscles |
| L/R triceps | L/R Pectoralis | Muscles of the leg |  |
| L/R deltoid | L/R Gastrocnemius | Muscles of the foot |  |

**S4 Table**. **Tissue property assignment for the tissues for which no measurements have been published**. Tissue categorization rule is followed by Virtual Population (15) and MIDA head model (16). Also, see **Table 6** for the converted tissue properties for 29-month-old (15–17).

| Database | Tissues in MARTIN |
| --- | --- |
| Adrenal Gland | Adrenal Gland |
| Air | Air Head, Air Neck |
| Bile | Bile |
| Blood | Blood, Heart Lumen |
| Blood Vessel Wall | Penis, Ureter, Urethra |
| Bone (Cortical) | Bone (Cortical), Skull Cortical, Tooth, Rib and Vertebrae (Cortical) |
| Bone Marrow (Red) | Bone Marrow Red, Skull (Bone Marrow Red) Vertebrae and Rib (Bone Marrow Red) |
| Brain (Grey Matter) | Brain (Grey Matter), Pallidum, Accumbens Area, Substantia Nigra, Amygdala, Mammillary body, Caudate, Putamen, Eye (Retina), Hippocampus, Hypothalamus, Thalamus |
| Brain (White Matter) | Brain White Matter, Cerebellum White Matter, Vermis White Matter |
| Cartilage | Nasal Cartilage, Long bones joint and femur cartilage, Secondary ossification centers in long bones |
| Cerebellum | Cerebellum, Medulla Oblongata, Midbrain, Pons, Vermis Grey Matter |
| Cerebrospinal Fluid | Cerebrospinal Fluid, CSF Spinal Cord, Eye (Aqueous Humor) |
| Dura | Meninges Brain, Meninges Spinal Cord |
| Esophagus | Esophagus |
| Eye (Aqueous Humor) | Eye Aqueous Humour |
| Eye (Cornea) | Eye (Cornea) |
| Eye (Lens) | Eye (Lens) |
| Eye (Sclera) | Eye (Sclera) |
| Eye (Vitreous Humor) | Eye (Vitreous Humor) |
| Fat (Average Infiltrated) | Fat, Subcutaneous Fat (SAT) |
| Gallbladder | Gallbladder |
| Heart Muscle | Heart Muscle |
| Intervertebral Disc | Intervertebral Disc |
| Intestine contents (10) | Large Intestine contents, Small intestine contents |
| Kidney | Kidney |
| Large Intestine | Large Intestine |
| Liver | Liver |
| Lung | Lung |
| Muscle | Muscle, Muscle periocular, Eye muscle, Mucous Membrane |
| Nerve | Optic nerves, Cranial Nerves (Large Branches II V VIII IX), Spinal Cord |
| Pineal Body | Pineal gland |
| Salivary Gland | Salivary Gland |
| Skin | Skin |
| Small Intestine | Small Intestine |
| Spleen | Spleen |
| Stomach | Stomach, Esophagus |
| Tendon\Ligament | Connective Tissue, Tendon\Ligament |
| Testis | Epididymis, Prostate, Seminal vesicle, Testis |
| Thymus | Thymus |
| Thyroid gland | Hypophysis, Pancreas, Pineal Body, Thyroid Gland |
| Tongue | Tongue |
| Trachea | Bronchi, Trachea |
| Trachea Lumen | Trachea Lumen |
| Urinary Bladder Wall | Bladder Wall |
| Urine | Urine |
| Uterus | Uterus |

**S5 Table**. *Uncertainty analysis. The methods used were based on the work of Neufeld et al.* (18) *to evaluate the uncertainty of the quantities derived by simulation, two simulations were assessed for each parameter by assigning two different values (“Value 1” and “Value 2”). The first value (“Value1”) was the one used for the simulation shown in* ***Table 7****, whereas the second value (“Value2”) was set to a realistic value that could occur from measurement or design choice. The results obtained for each value (“Result1” and “Result 2”) were used to evaluate the sensitivity factor of the quantity evaluated (10g averaged rms E-field). The standard deviation (“Std. Dev.”) was derived from literature.*

| Parameter | Quantity Evaluated | Value 1 | Value 2 | Result 1 | Result 2 | Sensitivity Factor | Std. Dev. (18) | Uncertainty (%) |
| --- | --- | --- | --- | --- | --- | --- | --- | --- |
| Cortical bone Conductivity | Maximum 10gE_wb_ [W/kg] | 0.14 | 0.13 | 281.05 | 299.66 | 0.62 %/% | 0.04 | 19.00 |
| Cortical bone Permittivity | Maximum 10gE_wb_ [W/kg] | 33.20 | 29.88 | 281.05 | 299.65 | 0.66 %/% | 2.8 | 5.58 |
| Cortical bone Density | Maximum 10gE_wb_ [W/kg] | 1908.00 | 1674.17 | 281.05 | 281.05 | 0.00 %/% | 10.00 | 0.00 |
| Muscle Conductivity [S/m] | Maximum 10gE_wb_ [W/kg] | 0.96 | 0.87 | 281.05 | 300.72 | 0.70 %/% | 0.04 | 2.98 |
| Muscle Permittivity | Maximum 10gE_wb_ [W/kg] | 85.24 | 76.72 | 281.05 | 298.49 | 0.62 %/% | 2.8 | 2.04 |
| Muscle  Density | Maximum 10gE_wb_ [W/kg] | 1090.40 | 1071.89 | 281.05 | 281.15 | 0.021 %/% | 10.00 | 0.019 |
| Nerve Conductivity [S/m] | Maximum 10gE_wb_ [W/kg] | 0.44 | 0.39 | 281.05 | 299.80 | 0.67 %/% | 0.04 | 6.26 |
| Nerve Permittivity | Maximum 10gE_wb_ [W/kg] | 67.18 | 60.46 | 281.05 | 299.64 | 0.66 %/% | 2.8 | 2.76 |
| Nerve Density | Maximum 10gE_wb_ [W/kg] | 1075.00 | 1056.70 | 281.05 | 281.04 | 0.0021 %/% | 10.00 | 0.0020 |
| Skin Conductivity | Maximum 10gE_wb_ [W/kg] | 0.65 | 0.59 | 281.05 | 303.43 | 0.80 %/% | 0.04 | 4.99 |
| Skin Permittivity | Maximum 10gE_wb_ [W/kg] | 118.90 | 107.00 | 281.05 | 299.05 | 0.64 %/% | 2.8 | 1.51 |
| Skin Density | Maximum 10gE_wb_ [W/kg] | 1109.00 | 1090.20 | 281.05 | 281.04 | 0.0025 %/% | 10.00 | 0.0023 |
| Position X of voxel model in Coil [mm] | Maximum 10gE_wb_ [W/kg] | 0 | 10 | 281.05 | 302.74 | 0.77 %/mm | - | - |
| Position Y of voxel model in Coil [mm] | Maximum 10gE_wb_ [W/kg] | 0 | 10 | 281.05 | 282.29 | 0.04 %/mm | - | - |
| Position Z of voxel model in Coil [mm] | Maximum 10gE_wb_ [W/kg] | 0 | -10 | 281.05 | 272.53 | 0.30 %/mm | - | - |

**S6 Table.** Skin depth calculation of 29-month-old child tissues in 1.5 T

| 1.5 T (64 MHz) | $\boldsymbol{\sigma}$ (S/m) | $\boldsymbol{\mu}$ (H/m) | $\boldsymbol{\delta}$ (m) |
| --- | --- | --- | --- |
| Skin | 0.65 | $4\pi\times{10}^{-7}$ | 0.08 |
| Muscle | 0.96 | $4\pi\times{10}^{-7}$ | 0.06 |

**S7 Table**. *Comparison of* s*pecific absorption rate and 10g averaged rms E-field results of Nina and MARTIN at 1.5T using Tx/Rx head coil using unadjusted (adult) tissue properties from IT’IS database* (17)

|  |  | Nina in 1.5 T using Head Tx/Rx coil  (Head centered, No implant) | MARTIN in 1.5T using Head Tx/Rx coil  (Head centered, No implant) |
| --- | --- | --- | --- |
| Fields were averaged to 2µT at the center of the coil (5) | Head averaged SAR (W/kg) | 0.15 | 0.14 |
|  | Whole-body averaged SAR (W/kg) | 0.03 | 0.03 |
|  | Whole-body maximum 10gSAR (W/kg) | 0.36 | 0.39 |
|  | Normalization factor (V) | 13.04 | 13.18 |
| Fields were averaged to 3.2 W/kg in the head  (Scanner head scanning limit) (2) | Maximum 10g rms  E-field (V/m) | 257.38 | 230.29 |
|  | Normalization factor (V) | 61.13 | 62.11 |

| **S1 Fig**. **Example of E_tan_ analysis on MARTIN model that can be used for the MRI RF safety of AIMD (ISO/TS 10974:2018 Tier-3)**. The results of E_tan_ can be used to assess RF safety of elongated medical devices by combining simulated incident E_tan_ on arbitrary lead trajectories using MARTIN model and measured response of AIMD on tissue simulating media. (a) magnitude of E_tan_ was calculated on MARTIN model along the arbitrary lead trajectories, (b) random possible lead trajectories were created around the potentially available routes of the lead using IMSAFE tool in Sim4Life, (c) E_tan_ from 20 routings were averaged at intervals of 5 mm (2), which can be used for estimating power deposition with a validated transfer function derived from an experimental test of AIMD on tissue simulating media. |
| --- |
| **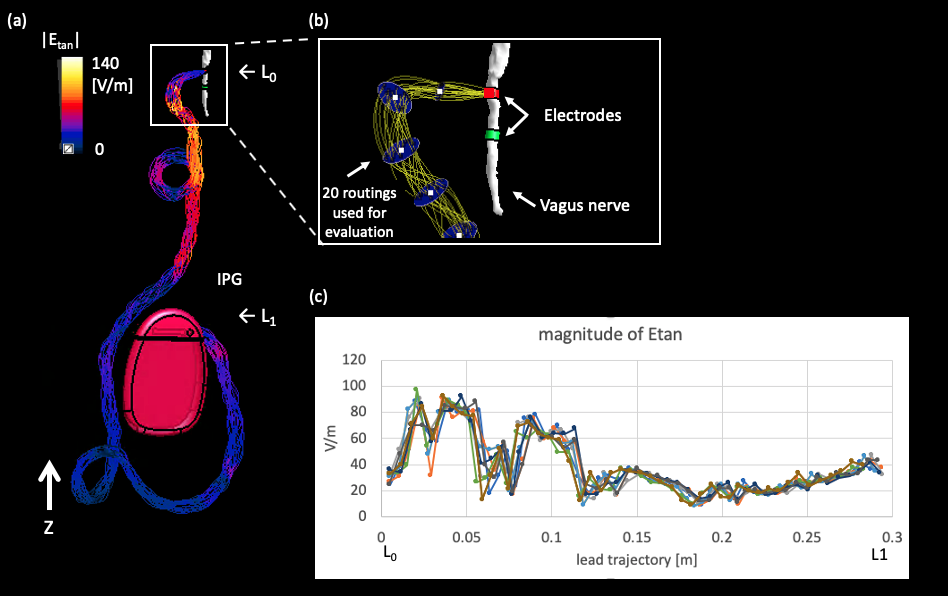** |
|  |

| **S2 Fig**. **The hand bone age validation. A different view of the left hand.** a) reference left-hand bone image; Reprinted from (19) under a CC BY license, with permission from Springer Nature, original copyright 2012. b) anterior view of the left hand in MARTIN, c) posterior-lateral view of the left hand in MARTIN, d) posterior view of the left hand in MARTIN |
| --- |
| 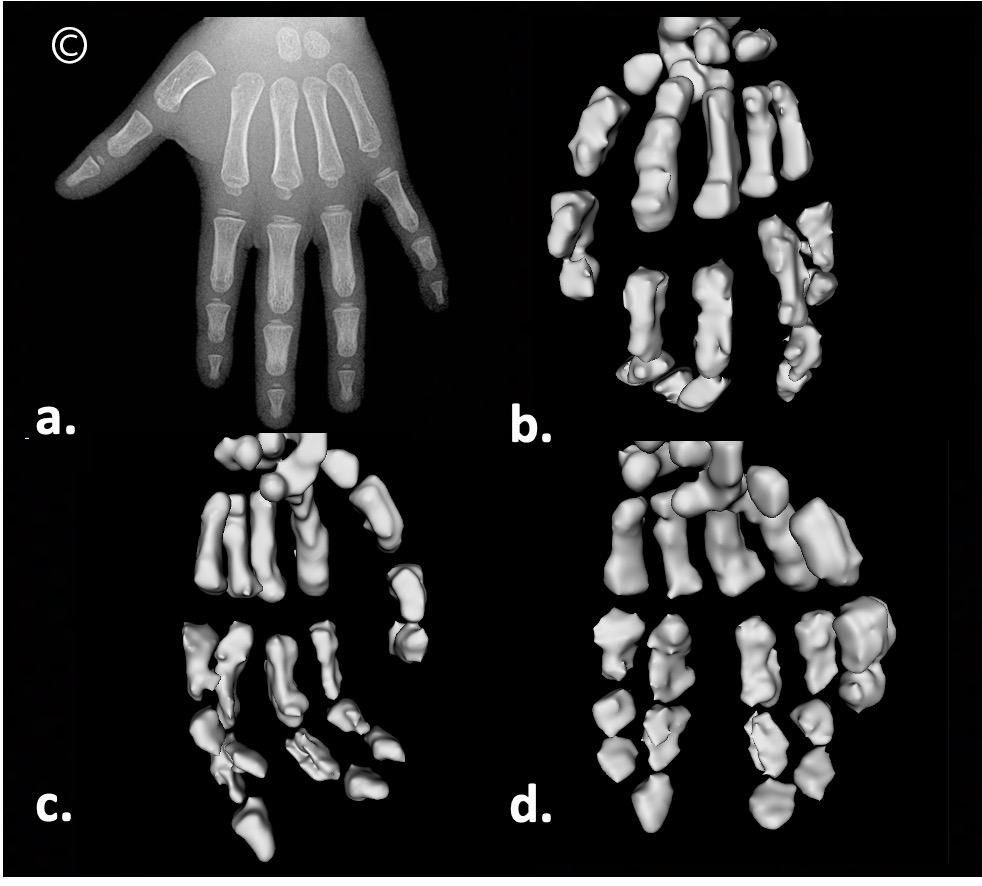 |

| **S3 Fig**. **Cross-correlation plots.** a) auto-correlation results of IR sequence in 2D view, b) auto-correlation results of SYSSIM results in 2D view, c) cross-correlation results between IR-sequence vs. SYSSIM results in 2D view, d) auto-correlation results of IR sequence in mesh surface plot, e) auto-correlation results of SYSSIM results in mesh surface plot, f) cross-correlation results between IR-sequence vs. SYSSIM results in mesh surface plot. |
| --- |
| **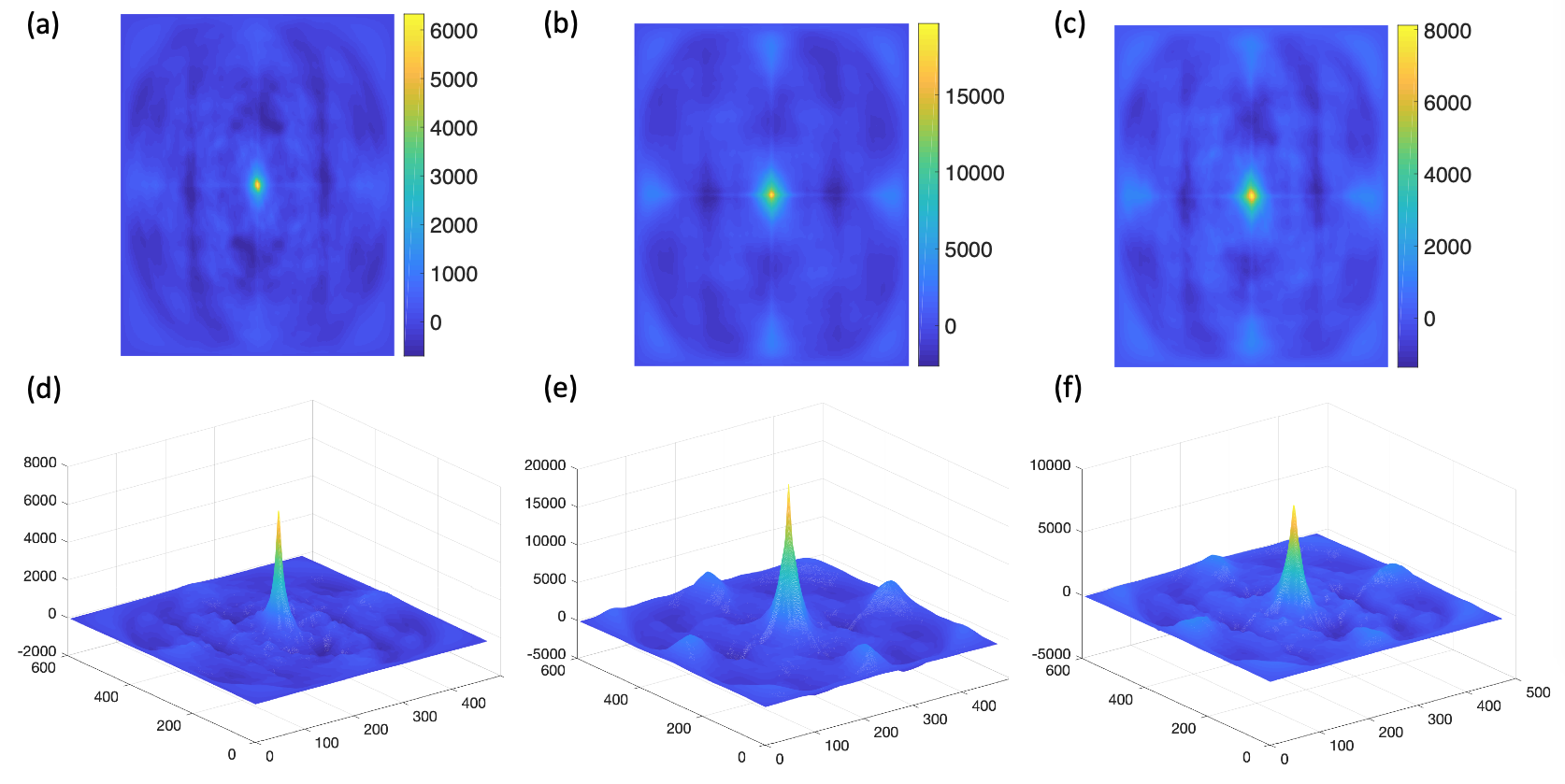** |

**References**

1. Makarov SN, Noetscher GM, Yanamadala J, Piazza MW, Louie S, Prokop A, et al. Virtual Human Models for Electromagnetic Studies and Their Applications. IEEE Rev Biomed Eng. 2017;10(99):95–121.

2. TS 10974:2018 Assessment of the safety of magnetic resonance imaging for patients with an active implantable medical device. 2nd ed. ISO/TC 150/SC 6. ISO; 2018.

3. Sim4Life by ZMT [Internet]. Available from: www.zurichmedtech.com

4. Fujita H, Zheng T, Yang X. A 3T Head Transmitter Integrated with 3D Parallel Imaging Capable 16-Channel Receive Array Coil. In: Proc Intl Soc Mag Reson Med. 2007. p. 3254.

5. IEC 60601-2-33:2010. IEC 60601-2-33:2010, Medical electrical equipment - Part 2-33: Particular requirements for the basic safety and essential performance of magnetic resonance equipment for medical diagnosis. International Electrotechical Commission; 2010.

6. Christ A, Kainz W, Hahn EG, Honegger K, Zefferer M, Neufeld E, et al. The Virtual Family—development of surface-based anatomical models of two adults and two children for dosimetric simulations. Phys Med Biol. 2010 Jan 21;55(2):N23–38.

7. Petoussi-Henss N, Zankl M, Fill U, Regulla D. The GSF family of voxel phantoms. Phys Med Biol. 2002;47(1):89–106.

8. Segars WP, Norris H, Sturgeon GM, Zhang Y, Bond J, Minhas A, et al. The development of a population of 4D pediatric XCAT phantoms for imaging research and optimization. Med Phys. 2015;42(8):4719–26.

9. Pi Y, Zhang L, Huo W, Feng M, Chen Z, Xu XG. Development and application of a set of mesh-based and age-dependent Chinese family phantoms for radiation protection dosimetry: Preliminary Data for external photon beams. EPJ Web Conf. 2017;153:1–6.

10. Malik SJ, Beqiri A, Price AN, Teixeira JN, Hand JW, Hajnal J V. Specific absorption rate in neonates undergoing magnetic resonance procedures at 1.5T and 3T. NMR Biomed. 2015;28(3):344–52.

11. Lee AK, Byun JK, Park JS, Choi H Do, Yun J. Development of 7-year-old Korean child model for computational dosimetry. ETRI J. 2009;31(2):237–9.

12. Lee C, Lodwick D, Hurtado J, Pafundi D, Williams JL, Bolch WE. The UF family of reference hybrid phantoms for computational radiation dosimetry. Phys Med Biol. 2010 Jan 21;55(2):339–63.

13. Henninger HB, Reese SP, Anderson AE, Weiss JA. Validation of computational models in biomechanics. Proc Inst Mech Eng Part H J Eng Med. 2010;224(7):801–12.

14. Zöllei L, Iglesias JE, Ou Y, Grant PE, Fischl B. Infant FreeSurfer: An automated segmentation and surface extraction pipeline for T1-weighted neuroimaging data of infants 0–2 years. Neuroimage. 2020 Sep;218:116946.

15. Gosselin M-C, Neufeld E, Moser H, Huber E, Farcito S, Gerber L, et al. Development of a new generation of high-resolution anatomical models for medical device evaluation: the Virtual Population 3.0. Phys Med Biol. 2014 Sep 21;59(18):5287–303.

16. Iacono MI, Neufeld E, Akinnagbe E, Bower K, Wolf J, Vogiatzis Oikonomidis I, et al. MIDA: A Multimodal Imaging-Based Detailed Anatomical Model of the Human Head and Neck. Jespersen SN, editor. PLoS One. 2015 Apr 22;10(4):e0124126.

17. PA H, F DG, C B, Neufeld E LB, MC G, D P, et al. IT’IS Database for thermal and electromagnetic parameters of biological tissues. IT’IS Foundation. IT’IS Foundation; 2018. Version 4.0.

18. Neufeld E, Kühn S, Szekely G, Kuster N. Measurement, simulation and uncertainty assessment of implant heating during MRI. Phys Med Biol. 2009 Jul 7;54(13):4151–69.

19. Gilsanz V, Ratib O, Spaltenstein J. Hand Bone Age: A Digital Atlas of Skeletal Maturity. Second Edi. Berlin, Heidelberg: Springer Berlin / Heidelberg. Berlin, Heidelberg: Springer Berlin / Heidelberg; 2011.
