# Supplementary material for: Development, validation, and pilot MRI safety study of a high-resolution, open source, whole body pediatric numerical simulation model
Source: PLoS One. 2021 Jan 13;16(1):e0241682. doi: 10.1371/journal.pone.0241682 (PMC7806143; doi:10.1371/journal.pone.0241682)
Supplement: Attachment — Submitted filename: Response to Reviewers2.docx [file pone.0241682.s002.docx]

5. Review Comments to the Author

Please use the space provided to explain your answers to the questions above. You may also include additional comments for the author, including concerns about dual publication, research ethics, or publication ethics. (Please upload your review as an attachment if it exceeds 20,000 characters)

Reviewer #1: This manuscript details the development of a ~3 year old child body model for computational modelling, including definition of tissue properties such as dielectric values, and a pilot study of a vagus nerve stimulating device included in the model and simulated for MRI safety. The phantom development is remarkable and the body model is of great interest to the research community. However, several issues with the manuscript dampen enthusiasm for publishing in its present form. Most concerns may be addressed straightforwardly by editing details in the manuscript. A couple comments are raised on technical aspects of the study. I sincerely hope the authors revise this manuscript and resubmit it soon so their commendable work may be utilized by the research community.

Major comments:
1-1. The limitations of existing child body models are arguably overstated in the Introduction. The Roberta model from the Virtual Population indeed is anatomically accurate. The only issue is that Roberta is based on images from a 5 year old, whereas the present study prefers a 3 year old.

*Thank you for this suggestion. We agree that the limitations of existing child body models are overstated in the Introduction, and the only issue of Roberta is based on images from a 5-year-old, whereas our proposed model is based on a 29-month-old. We simplified the limitation of current pediatric models and moved the detailed survey of pediatric models to the Supporting Information S2. (Section 1 Paragraph 1, Supporting Information S2).*

**Revised results*

*Following is the edited paragraph in the Introduction. ‘The most anatomically accurate child model to date is Roberta (21), which is a model of a 5-year-old female. However, all the 3-year-old models currently available are not anatomically accurate (see Table 1 and Supporting Information Section 2 for the complete survey of the existing pediatric models). For instance, Nina is a 3-year-old female model, and it was morphed from Roberta, which resulted in the anatomical inaccuracies due to the anisotropic growth of different tissues since the body tissues do not grow proportionally during childhood.' (Section 1:Introduction Paragraph 1)*

*'(We moved the limitations of existing child models from Introduction to the Supporting information) S2 Appendix* ***contains additional information on the existing pediatric numerical models****.*

*'‘Appendix*

*Various children models are introduced or morphed from an older age group, such as the Nina from the Virtual Family (6), the GSF family (Baby, Child) by Helmholtz Zentrum München (7), the pediatric xCAT phantom (8), and Chinese family (9). The Baby model in the GSF family is also used for the development of the Charlie model for Sim4Life users. However, these models have gaps in the validation of their segmentation anatomical details as the anatomy may contain inaccuracies due to the anisotropic growth of the different tissues, as shown in Table 1. These models are designed and used for neonatal numerical modeling (10), but not represent 29-month-old well. For example, a Baby model in the GSF family has an incomplete tissue segmentation of the brain (7), which is essential for implant RF safety assessment in MRI. A model with detailed structures will allow for the correct anatomical placement of implants and study the potential consequences or side effects of heating in the surrounding tissues. Furthermore, the currently available children numerical models younger than five have been morphed from older body models (7,8,11,12), which may produce anatomical inaccuracies (13) since the body tissues do not grow proportionally during childhood.'*

1.2 If the manuscript is to detail at length the inadequacies of currently-available child body models, then this study should compare simulation results from MARTIN with results using Nina (which was morphed from Roberta). Typically, an important measure of a new tool for the research community is a rigorous comparison with the next best existing tool.

*Thank you for the suggestion to compare the simulation results from MARTIN with the simulations using Nina. We made a rigorous comparison between MARTIN and Nina running RF safety simulation in 1.5 T using a head Tx/Rx coil, by comparing different SAR averages, 10g averaged E-field. The simulation results of Nina were performed using the same tissue properties (3-year-old tissue properties) that were used for Martin and were added in Table 7. In addition to the comparison of the results with 3-year-old tissue properties, we conducted additional simulations using unadjusted tissue properties (adult) from IT'IS database using both Nina and Martin and compared in Supporting Information Table-8. (Section 3 – Pilot Use Case for MRI RF safety Simulation, Table 7, S7 Table).*

**Revised results*

*Following is the edited paragraph in the discussion. ‘Finally, we compared the results of head averaged SAR from Martin with the results using Nina model (Table 7), which were 0.2046 W/kg and 0.2068 W/kg, corresponding only to marginal variations of 1.06 %. (See S7 Table for the simulation results comparison between Martin and Nina with adult tissue properties (23)). Even though the variations are marginal, the anatomical inaccuracies of Nina not present in MARTIN, may lead to SAR peaks in mis-labelled tissue.' (Table 7, Section 4 – Pilot Use Case for MRI RF safety Simulation)*

Excerpt from **'Table 7**. *Specific absorption rate and 10g averaged rms E-field results of Nina and MARTIN at 1.5T using Tx/Rx head coil '*

|  |  | Nina in 1.5 T using Head  Tx/Rx coil  (Head centered, No implant) | MARTIN in 1.5T using Head Tx/Rx coil  (Head centered, No implant) |
| --- | --- | --- | --- |
| Fields were averaged to 2µT at the center of the coil (89) | Head averaged SAR (W/kg) | 0.21 | 0.20 |
|  | Whole-body averaged SAR (W/kg) | 0.04 | 0.05 |
|  | Whole-body maximum 10gSAR (W/kg) | 0.48 | 0.49 |
|  | Normalization factor (V) | 13.71 | 13.95 |
| Fields were averaged to 3.2 W/kg in the head  (Scanner head scanning limit) (84) | Maximum 10g rms  E-field (V/m) | 176.54 | 165.45 |
|  | Normalization factor (V) | 53.94 | 55.17 |

**S7 Table**. *Comparison of* s*pecific absorption rate and 10g averaged rms E-field results of Nina and MARTIN at 1.5T using Tx/Rx head coil using unadjusted (adult) tissue properties from IT'IS database (18)’*

|  |  | Nina in 1.5 T using Head Tx/Rx coil  (Head centered, No implant) | MARTIN in 1.5T using Head Tx/Rx coil  (Head centered, No implant) |
| --- | --- | --- | --- |
| Fields were averaged to 2µT at the center of the coil (5) | Head averaged SAR (W/kg) | 0.15 | 0.14 |
|  | Whole-body averaged SAR (W/kg) | 0.03 | 0.03 |
|  | Whole-body maximum 10gSAR (W/kg) | 0.36 | 0.39 |
|  | Normalization factor (V) | 13.04 | 13.18 |
| Fields were averaged to 3.2 W/kg in the head  (Scanner head scanning limit) (2) | Maximum 10g rms  E-field (V/m) | 257.38 | 230.29 |
|  | Normalization factor (V) | 61.13 | 62.11 |

1-3. There is a brief mention of comparison to the neonatal model reported by Malik et al 2015 [109] however that model does not appear to be included in Table 1, nor are details of that model discussed.

*Lastly, the model used by Malik et al. 2015 was developed by Helmholtz Zentrum München it is now in Table1. The details of this model are in the Supporting Information S2. (Section 4-Pilot Use Case for RF Safety Simulation, Table 1, Supporting Information S2)*

**Revised results*

*Following is the edited paragraph in the discussion* ‘*SAR_head_, SAR_wb_, and 10gSAR in 1.5T reported in this study with MARTIN and Nina without an implant were consistent with that of the Baby model developed by Helmholtz Zentrum München (29) reported in 2015 by Malik et al. (111), which was a valuable reference to compare the simulation setup and use of dielectric properties conversion. (Section 4-Pilot Use Case for MRI RF Safety Simulation).*

*Following is the edited paragraph in the Supporting Information* ‘S2 Appendix **contains additional information on the e**xisting pediatric numerical models.

*‘‘Appendix*

*Various children models are introduced or morphed from an older age group, such as the Nina from the Virtual Family (6), the GSF family (Baby, Child) by Helmholtz Zentrum München (7), the pediatric xCAT phantom (8), and Chinese family (9). The Baby model in the GSF family is also used for the development of the Charlie model for Sim4Life users. However, these models have gaps in the validation of their segmentation anatomical details as the anatomy may contain inaccuracies due to the anisotropic growth of the different tissues, as shown in Table 1. These models are designed and used for neonatal numerical modeling (10), but not represent 29-month-old well. For example, a Baby model in the GSF family has an incomplete tissue segmentation of the brain (7), which is essential for implant RF safety assessment in MRI. A model with detailed structures will allow for the correct anatomical placement of implants and study the potential consequences or side effects of heating in the surrounding tissues. Furthermore, the currently available children numerical models younger than five have been morphed from older body models (7,8,11,12), which may produce anatomical inaccuracies (13) since the body tissues do not grow proportionally during childhood.’ (Supporting Information S2)*

2. SSIM is a perception-based model, while alternative objective assessment measures, e.g., mean squared error, quantitatively describe absolute errors. SSIM is well suited for describing the effectiveness of an image compression algorithm, e.g., would a radiologist still perceive the relevant features of a compressed image versus the uncompressed image (based on contrast groups, as the authors state)? With so much care directed to the precise tissue parameters, e.g., T1, T2, dielectric properties, etc., why base the analysis of the new body phantom with the Bloch simulator on SSIM measures instead of MSE? Is the goal of the Bloch simulator aspect of the study to determine if the body model will produce images that are perceived to be similar to actual MRI, or is the goal to gauge how well the body model and appropriate tissue properties are a theoretical match to actual MRI?

*Thank you for this suggestion. We agree that the SSIM is not an ideal assessment method to compare the original MR image with the synthetic MR image produced using MARTIN via the Bloch simulator. The goal of the Bloch simulator was to determine whether the segmented MARTIN model will produce images that are similar to actual MRI. Following the reviewer’s suggestion, the mean-squared-error (MSE) is now used instead of SSIM, which returned the value of 0.14 (Section 3 Result -Th Validation Paragraph 4). An additional assessment was conducted using cross-correlation between IR sequence and SYSSIM results. (S3 Fig 3)*

**Revised results*

*Following is the edited paragraph in the result section. ‘The matching between the MRI image and the SYSSIM results was assessed using the mean-squared-error (MSE) and the cross-correlation.’(Section 2 – The Validation Paragraph 2)*

*‘The MSE between the original IR sequence and SYSSIM returned the value of 0.14 (in normalized and zero averaged intensities), which along with the cross-correlation coefficient of 0.782 (See S3 Fig for the cross-correlation plots) indicates a high level of matching between the two images.’ (Section 3 Result -Th Validation Paragraph 4).*

**‘S3 Fig 3**. **Cross-correlation plots.** a) auto-correlation results of IR sequence in 2D view, b) auto-correlation results of SYSSIM results in 2D view, c) cross-correlation results between IR-sequence vs. SYSSIM results in 2D view, d) auto-correlation results of IR sequence in mesh surface plot, e) auto-correlation results of SYSSIM results in mesh surface plot, f) cross-correlation results between IR-sequence vs. SYSSIM results in mesh surface plot.’

Minor comments:
3. In the Materials and Methods subsection on The Validation, calculation of tissue compartment density is discussed. Then, in the Dielectric properties subsection there is a rigorous discussion of the change of dielectric properties with age. Does the literature suggest any variations in tissue density based on age? If so, perhaps this should also be considered in this study.

*Thank you for the reviewer’s comment. We agree that the variations in tissue density based on age were not estimated in the original manuscript since we followed the study of Malik et al. We searched PubMed for articles on age-dependent density variation of the different tissues. We found that the tissue densities are only minimally sensitive to aging with a total variation of up to 1.7%* (14)*. Therefore, we studied this variation and reported the results in the uncertainties table, which confirmed that the tissue densities changes with age can be neglected. (Section 3 – Pilot Use Case for MRI RF Safety Simulation, S5 Table)*

**Revised results*

*Following is the added paragraph in the materials and method section. ‘According to J. Wells et al., the variation of tissue density with age is relatively small for lean tissues (1.7 % lower for the 5-year-old than adult lean tissue density) (82). The tissue densities used in our simulations are from the tissue density database in IT’IS (23).’ (Section 2 – The validation)*

*Following is the added paragraph in the result section. ‘We studied the tissue density variation of 1.7 % in the uncertainties table in Supporting Information (S5 Table), and found that the uncertainty budget is very small, < 0.03 %/%, thus can be neglected (36) (Section 3 – Pilot Use Case for MRI RF Safety Simulation)*

Excerpt from **‘S5 Table**. *Uncertainty analysis. The methods used were based on the work of Neufeld et al. (18) to evaluate the uncertainty of the quantities derived by simulation, two simulations were assessed for each parameter by assigning two different values (“Value 1” and “Value 2”). The first value (“Value1”) was the one used for the simulation shown in* ***Table 7****, whereas the second value (“Value2”) was set to a realistic value that could occur from measurement or design choice. The results obtained for each value (“Result1” and “Result 2”) were used to evaluate the sensitivity factor of the quantity evaluated (10g averaged rms E-field). The standard deviation (“Std. Dev.”) was derived from literature.’*

| Parameter | Quantity Evaluated | Value 1 | Value 2 | Result 1 | Result 2 | Sensitivity Factor | Std. Dev. (18) | Uncertainty (%) |
| --- | --- | --- | --- | --- | --- | --- | --- | --- |
| Cortical bone Density | Maximum 10gE_wb_ [W/kg] | 1908.00 | 1674.17 | 281.05 | 281.05 | 0.00 %/% | 10.00 | 0.00 |
| Muscle  Density | Maximum 10gE_wb_ [W/kg] | 1090.40 | 1071.89 | 281.05 | 281.15 | 0.021 %/% | 10.00 | 0.019 |
| Nerve Density | Maximum 10gE_wb_ [W/kg] | 1075.00 | 1056.70 | 281.05 | 281.04 | 0.0021 %/% | 10.00 | 0.0020 |
| Skin Density | Maximum 10gE_wb_ [W/kg] | 1109.00 | 1090.20 | 281.05 | 281.04 | 0.0025 %/% | 10.00 | 0.0023 |

4. The Pilot simulation study is unclear regarding the type of solver in Sim4Life that was used. Was it FEM or FDTD? It seems it was FEM, since much attention is paid to the surface mesh extraction and unstructured tetrahedral mesh surfaces, however it remains unclear.

*Thank you for this query. Authors agree that the simulation solver used for Pilot simulation was unclear in the original manuscript. We used the finite-difference time-domain (FDTD) method that is implemented in the commercial software Sim4Life. The simulation solver used in the Pilot simulation was added in Section 2 Materials and Methods – The Pilot Use Case for MRI RF Safety Simulation.*

**Revised results*

*Following is the edited paragraph in the materials and method section. ‘An MRI RF safety simulation was performed on MARTIN by implanting VNS electrodes to illustrate how safety simulation studies may be conducted. The simulation consisted of estimating the electric field (E-field) distribution and the specific absorption rate (SAR). Examples of simulations followed the newest (2018) International Organization for Standardization Technical Specification (ISO/TS) 10974 – assessment of the safety of magnetic resonance imaging for patients with an active implantable medical device (83). Sim4Life (ZMT, Switzerland) was used to solve the Maxwell equation at 64 MHz using the finite-difference time-domain (FDTD) method (84)’ (Section 2 Materials and Methods – The Pilot Use Case for MRI RF Safety Simulation Paragraph 1)*

5. The Introduction motivates the study by reviewing the lack of validated child body models based on child image sets, as well as the safety questions surrounding MRI of children with VNS. Since it is mentioned PubMed does not presently include studies focusing on MRI safety of children with VNS, it would be beneficial to include a brief survey of existing MRI safety studies for adults with VNS. The one reference in the manuscript is Shellock et al. [43], which was published 16 years ago. I would suggest adding more recent literature to the Introduction to motivate this child body model and simulation project. Examples include de Jonge et al. 2014 (doi: 10.1111/epi.12774), which included 11 patients between 5-12 years old, Gorny et al. 2010 (doi: 10.1002/jmri.22037), and Rösch et al. 2015 (doi: 10.1016/j.eplepsyres.2014.11.010).

*Thank you for this suggestion. We agree that the study done by Shellock et al. was conducted more than 10 years ago, thus it may not be sufficient to motivate the needs for the child body model uses in VNS RF safety simulation. Authors appreciate the reviewer’s suggestion to add more recent studies of existing MRI safety for adults and children with VNS age between 5-12 years old to the Introduction. We added suggested references to the Introduction, and we believe it strengthens the motivation of the proposed voxel model study. (Section 1 – Introduction Paragraph 3)*

**Revised results*

*Following is the added paragraph in the Introduction section. ‘Several clinical studies conducted at 1.5 T and 3T have shown that adults (43,44) and younger subjects with ages between 5 and 12 (45) can be scanned in MRI under controlled condition using a transmit/receive head coil without any report of heating, discomfort, or any other unusual sensation. The numerical simulations of MRI RF safety of pediatric subject with VNS are not presented in literature yet.’ (Section 1 – Introduction Paragraph 3)*

6. In the Introduction, Wolf et al. [34] is cited to substantiate the argument that low-quality models can lead to SAR underestimation. Wolf referred to the original Virtual Family models not including CSF in the brain, thus removing the highest conductivity tissue from the head; CSF was added in later versions. This does not exactly substantiate the argument that a different sized organ, e.g., the heart, will lead to underestimation, even if that argument is correct.

*Thank you for this suggestion. We agree that the study done by Wolf et al. does not substantiate the argument that a different sized organ will lead to underestimation. We changed that sentence with the sentence that substantiates the improved SAR exposure estimation with the model resolution.*

**Revised results*

*Following is the modified sentence in the Introduction. ‘Furthermore, the higher level of detail in the high-resolution model led to a better estimation of the SAR exposure in the vertebrae, where the layer of cortical/cancellous bones can clearly be distinguished; by comparison, a low-resolution model included only one type of bone in the vertebrae (21).’ (Section 1 – Introduction Paragraph 1)*

7-1. The authors discuss the advantage of this study is the use of in vivo medical images of a 29-month-old child and the age appropriateness of the model tied to the subject’s age. However, given the CT data were acquired when the boy was 24 months old, the gap and required deformation seems to undercut part of the stated motivation for this study (not morphing or adjustments).

Thank you for this observation. We agree that the age of the CT data has 5-month gaps compared to the MR data. The MR and CT images used for the segmentation were clinical recordings obtained from the PACS system of Boston Children’s Hospital. It is not common for a child without a distinctive disease that could affect its anatomy have whole-body MRI and also CT scans at the same time. This remains a limitation of our study (stated in Section 4 – Discussion and Conclusion – The Validation) which is based on clinical data. This is probably the reason why most of the pediatric models are segmented either from an MRI or a CT alone (Table 1). In our model, we used the MRI scans to segment most of the bones, such as the Radius, Tibia, Ulna, and many others (shown in S2 Table). The morphed CT data were used to initially segment the mandible, the vertebrae, and the rib cage. All of this initial segmentation was reevaluated and modified on based on the reference MR data as the CT data were not perfectly aligned in a few spots due to the different time of the scan, the posture of the subject, and the respiration state (e.g., misalignment of the ribcage around the lungs). The limitation of the CT data is added to the discussion. (Section 4 – Discussion and Conclusion – Limitation on segmentation).

**Revised results*

*Following is the edited paragraph in the Discussion and Conclusion ‘Also, the CT data has a 5-month time lag compared to the MR data as the MR and CT images used for the segmentation were clinical recordings obtained from the PACS system of Boston Children’s Hospital. We could not find in our PACS system a patient without a distinctive disease that could affect its anatomy to have whole-body MRI and also CT scans at the same time. The CT data were morphed to aid manual segmentation of the mandible, the vertebrae, and the rib cage, while the MRI scans were used to segment most of the bones, such as the Radius, Tibia, Ulna, and many others (see S2 Table). All of this initial segmentation was reevaluated and modified on based on the reference MR data as the CT data were not perfectly aligned in a few spots due to the different time of the scan, the posture of the subject, and the respiration state (e.g., misalignment of the ribcage around the lungs).’ (Section 4 – Discussion and Conclusion – Limitation on segmentation Paragraph 1).*

7-2. On a second note, given 29 months is closer to 2 years compared to 3 years, perhaps the model should not be described as a three year old child.

*Thank you for this suggestion. Authors agree that 29 months is closer to 2 years compared to 3 years, thus it is better not to describe the model as a three-year-old. The age of the model described in the manuscript is changed to 29-month-old (Section 2 – Method: Dielectric properties, Section 3 Results – Pilot Use Case for MRI RF Safety Simulation, Section 4 – Discussion and Conclusion, Table 6, S4 Table, and S6 Table)*

8. The reference list needs to be carefully reviewed for formatting errors. [6] and [83] incorrectly abbreviate organization or document names as if they were a given name and surname. [23] misspells the first author’s surname. [42] does not provide enough information to be useful. [81] does not include a publication year.

*Thank you for this suggestion. We agree that the reference list should be carefully reviewed for formatting errors. Reference [6] and [83] are updated with the correct abbreviated organization. Also, for the [23],[42], and [81].*

**Revised results*

*Following is the edited references in the reference section.*

*[6] ® [6] Reporting of Computational Modeling Studies in Medical Device Submissions - Draft Guidance for Industry and Food and Drug Administration Staff. Vol. 79, The Federal Register / US FDA. 2016. 3211 p.*

*[83] ® [83] TS 10974:2018 Assessment of the safety of magnetic resonance imaging for patients with an active implantable medical device. 2nd ed. ISO/TC 150/SC 6. ISO; 2018.*

*[23] ® [23] PA H, F DG, C B, Neufeld E LB, MC G, D P, et al. IT’IS Database for thermal and electromagnetic parameters of biological tissues. IT’IS Foundation. IT’IS Foundation; 2018. Version 4.0.*

*[42] ® [40] LivaNova Celebrates 25 Years of Vagus Nerve Stimulation Therapy [Internet]. LivaNova PLC. Available from:* [*https://investor.livanova.com/news-releases/news-release-details/livanova-celebrates-25-years-vagus-nerve-stimulation-therapy*](https://investor.livanova.com/news-releases/news-release-details/livanova-celebrates-25-years-vagus-nerve-stimulation-therapy)

*[81] ® [80] Peyman A. Mechanisms of Interaction Electromagnetic of Radiation with Biological Tissues. University of London; Doctoral Thesis; 2002.*

9. Table 1 indeed lists “models currently available” however they are all not necessarily “state-of-art” unless they were released in recent years. If some are older, I suggest removing “state-of-art”. However, perhaps the table should include the year each model was released, as that provides additional valuable information.

*Thank you for this suggestion. We agree with the reviewer’s point that all models are not necessarily “state-of-art” unless they were released in recent years. The “state-of-art” is removed from Table 1, and the year of the model released is now added as additional valuable information in Table 1.*

**Revised results*

*Following is the edited Table 1.*

Excerpt from *‘***Table 1**. *List of pediatric whole-body models currently available.’ (Table 1)*

| Model Name | Charlie | Nina | Roberta | Korean Child model  ETRI | UF Family of reference hybrid phantoms | GSF Family  (Baby,Child) | Pediatric xCAT phantoms | Chinese family phantom USTC |
| --- | --- | --- | --- | --- | --- | --- | --- | --- |
| Year of model released | **2014** | **2014** | **2014** | **2009** | **2010** | **2003** | **2015** | **2017** |

10. Table 1 states the Virtual Population models Charlie, Nina, and Roberta are not freely accessible to the research community. In fact, V1.x of those models are available to the academic community for a nominal shipping and handling charge for the data DVD.

Thank you for this observation. We contacted the IT’IS office to clarify the availability and information of their pediatric models. According to IT’IS representative, “The 4 models of the Virtual Classroom (Roberta, Louis, Dizzy and Eartha) are not any more distributed free of charge for academic use. In this case, for the voxel format of these V1.x models, a license fee also applies for academic use. The same would apply to Nina and Charlie in voxel format. The V1.x models currently available free of charge for academic use (shipping and handling fees excluded) are only the 4 models of the Virtual Family (Duke, Ella, Billie, Thelonious). These four models are distributed on a DVD, which includes the models in both CAD format (.sab, compatible only with the simulation software SEMCAD X and Sim4Life) and in voxel format (.raw, open to any compatible FDTD-based solver).” For this reason, we obtained a one-month trial license of Nina to follow-up on revision. We were also advised by IT’IS to label the imaging modality used for ‘Charlie’ and ‘Nina’ models to Baby model by Helmholz Zentrum München and Roberta, respectively. The imaging modality used for Roberta is updated to MRI by correction IT’IS. (Table. 1)

**Revised results*

*Following is the edited column in Table 1.*

Excerpt from **‘Table 1**. *List of pediatric whole-body models currently available.*’

| Model Name | Charlie | Nina | Roberta |
| --- | --- | --- | --- |
| Year of model released | **2014** | **2014** | **2014** |
| Imaging modalities used | GSF family  Baby (29) | Roberta  (21) | MRI |
| Age imaging scans used for segmentation | Eight-week-old | 3-year-old | 5-year-old |
| Number of tissue varieties | 45 tissues | 75 tissues | 78 tissues |
| Segmented both white- and grey-matter | Yes | Yes | Yes |
| Morphing or adjustments used | No | Yes | No |
| Method of validation | Anatomical  knowledge | Anatomical  knowledge | Anatomical  knowledge |
| Is the model freely accessible to the research community | No | No | No |
| References/  company | Petoussi-Henss *et al.* (29) /  IT’IS (21) | IT’IS (21) | IT’IS (21) |

11. Table 1 states "Information not available" for two of the phantoms. Did the authors inquire as to the availability of these phantoms? I know this is not a journalism manuscript, but still some may consider it worthwhile to ask to ascertain details before reporting there is no available information.

*Thank you for this observation. We contacted all model developers and updated them in Table 1. Although the developer of the Chinese family is not responding to our request even after multiple requests, we remained section as not available.*

**Revised results*

*Following is the edited texts in Table 1.*

**‘Table 1**. *List of pediatric whole-body models currently available.*’

| Model Name | Charlie | Nina | Roberta | Korean Child model  ETRI | UF Family of reference hybrid phantoms | GSF Family  (Baby,Child) | Pediatric xCAT phantoms | Chinese family phantom USTC |
| --- | --- | --- | --- | --- | --- | --- | --- | --- |
| Year of model released | **2014** | **2014** | **2014** | **2009** | **2010** | **2003** | **2015** | **2017** |
| Imaging modalities used | GSF family, Baby (29) | Roberta  (21) | MRI | MRI | CT | CT | PET-CT | RPI mesh phantom (30) |
| Age imaging scans used for segmentation | Eight-week-old | 3-year-old | 5-year-old | 7-year-old | Newborn, 1-, 5- and 10-year- old | Eight-week-old and 7-year-old | Newborn,1-,5-,10-,15-year-old | 5-, 10-,15-year-old |
| Number of tissue varieties | 45 tissues | 75 tissues | 78 tissues | 44 tissues | 47 tissues | 41 tissues | 64 tissues | 50 tissues |
| Segmented both white- and grey-matter | Yes | Yes | Yes | Yes | No | No | No | No |
| Morphing or adjustments used | No | Yes | No | Yes | Yes | Yes | Yes | Yes  Database:  50^th^ percentile population |
| Method of validation | Anatomical  knowledge | Anatomical  knowledge | Anatomical  knowledge | Comparison with the physiques measurement of reference models | Comparison with the physiques measurement of reference models | Comparison with the physiques measurement of reference models | Comparison with the physiques measurement of reference models | Comparison with the physiques measurement of reference models |
| Is the model freely accessible to the research community | No | No | No | No | Yes, upon request | Yes, upon request | No | Information not available |
| References/  company | Petoussi-Henss *et al.*, 2002 (29) /  IT’IS (21) | IT’IS (21) | IT’IS (21) | Lee *et al*., 2009 (31) | Lee *et al*., 2010 (32) | Petoussi-Henss *et al.* 2002 (29) / Helmholtz Zentrum München | Segars *et al*., 2015 (33,34) | Pi *et al*., 2017 (35) |

12. To improve readability, the manuscript would benefit from careful editing by a native English speaker. Some changes suggested by this reviewer follow:

*Thank you for your suggestion. We followed the reviewer’s suggestion of correction. Following is the updated text in each section as below, plus careful review from all the authors.*

- Intro: change "computational with human body modeling" to "computational modeling with the human body"

*The areas of computational modeling with human body are wide: fluid dynamics (1), electromagnetics (2), optics (3), ultrasound (4), thermodynamics (5), and mechanics (6,7). Computational modeling with virtual humans is helpful in studying the interaction of complex biological problems in silico (7), for source localization (8,9), radio-frequency (RF) and specific absorption rate (SAR) exposure (10), and neurostimulation (11–13).*

- Intro: "electromagnetism (2)" to "electromagnetics (2)" (note how other areas listed tend to end with -ics)

*The areas of computational modeling with human body are wide: fluid dynamics (1), electromagnetics (2), optics (3), ultrasound (4), thermodynamics (5), and mechanics (6,7). Computational modeling with virtual humans is helpful in studying the interaction of complex biological problems in silico (7), for source localization (8,9), radio-frequency (RF) and specific absorption rate (SAR) exposure (10), and neurostimulation (11–13).*

- Intro: "computational tomography" should be "computed tomography"

*The accurate anatomical representation of human numerical models has become an integral part of many state-of-the-art safety studies, such as computed tomography (CT) dosimetry (14) and in MRI RF exposure (15–17). The Visible Photographic Man (VIP-MAN) is a well-known, image-based whole-body model that was introduced by the National Library of Medicine’s Visual Human Project (18,19).*

- Intro: "segmented into 3D whole-body voxel model" should be "segmented into a 3D ..."

*The cadaver of a 38-year-old male was imaged with four data modalities; X-ray, CT, MRI, and cryosection color photography, and the data were segmented into a 3D whole-body voxel model.*

- Intro: Virtual Family should have capitalized first letters

*More recently, the Virtual Family was introduced with various ages ranging between eight weeks and 80 years by IT’IS (20,21).*

- Intro: "various ages ranging between an eights-weeks and an 80-years-old" should be "various ages ranging between eight weeks and 80 years" (grammatically, please note the age should be hyphenated if used as a compound modifier, but otherwise not hyphenated)

*More recently, the Virtual family was introduced with various ages ranging between eight weeks and 80 years by IT’IS (20,21).*

- Intro: "using images from a whole body in-vivo subject." should be "using images from whole body in-vivo subjects." if you are referring to all the Virtual Family and not just one model. If you are only referring to one model in the Virtual Family, then it is correct as written but the preceding sentence should be modified as it refers to multiple Virtual Family models.

*The segmentation of tissue compartments was done using medical images from whole-body in-vivo subjects.*

- Intro: "neck model is MIDA" should be "neck models is MIDA"

*By far, one of the most anatomically accurate head and neck models is MIDA (16), which is an open-source model that includes the 115 structures with individual nerve tracts and deep brain structures, and it was used for estimating the MR signal intensities (24), neuronal stimulation (25,26), and the assessment of electromagnetic (EM) field distribution in the head (27).*

- Intro: "do not grow symmetrically" -- do the authors mean "proportionally"? "Symmetrically" implies left-right symmetry as opposed to different organs growing proportionally.

*For instance, Nina is a 3-year-old female model, and it was morphed from Roberta, which resulted in the anatomical inaccuracies due to the anisotropic growth of different tissues since the body tissues do not grow proportionally during childhood.*

- Intro: "it is a 5-year-old model" -- is this referring to the age of the model (present writing implies this) or referring to the age of the child that is modeled? If the latter, suggest "is a model of a 5 year old".

*The most anatomically accurate child model to date is Roberta (21), which is a model of a 5-year-old female.*

- Intro: "66 tissue segmentation" to "66 tissues segmented"

*The description of “66 tissue segmentation” is deleted as corresponding information is not necessary to repeat. (already described in Table 1).*

- Intro: "Having both more tissues are visible for accurate segmentation" -- please rewrite, the message is confusing as written.

*The segmentation was done based on in-vivo MRI and CT data, which allow accurate tissue segmentation for a 29-month-old child.*

- Intro: "includes 86 tissue segmentation" to "includes 86 segmented tissues"

*In summary, we have introduced MARTIN, a 29-month-old boy whole-body model, which includes 86 segmented tissues, segmented directly from MRI and CT images of a 29-month-old child, and was validated using three separate approaches.*

- Methods: suggest standardizing to the vendor and/or scanner model being all caps or not all caps, e.g., SIEMENS TrioTim, Siemens SOMATOM, Siemens Healthinears, SIEMENS Healthinears.

*The MRI data included both T1 and T2 weighted sequences were acquired with a SIEMENS TRIOTIM 3T scanner (SIEMENS Healthineers, Erlangen, Germany) from the top of the skull through the toes (Table 2). The CT scans were acquired on a SIEMENS SOMATOM DEFINITION FLASH (SIEMENS Healthineers, Erlangen, Germany) that covered the partial-head and neck. (Table 2)*

- Methods: regarding the above sentences, note the typos "Healthinears" should be "Healthineers"

*The MRI data included both T1 and T2 weighted sequences were acquired with a SIEMENS TRIOTIM 3T scanner (SIEMENS Healthineers, Erlangen, Germany) from the top of the skull through the toes (Table 2). The CT scans were acquired on a SIEMENS SOMATOM DEFINITION FLASH (SIEMENS Healthineers, Erlangen, Germany) that covered the partial-head and neck. (Table 2)*

- Methods: suggest describing 3D Slicer as "an open-source software platform" at the first mention of 3D Slicer in the Data co- registration subsection.

*Data co-registration: Co-registration between different sequences and modalities was done using an extension tool in 3D Slicer, an open-source software platform (46).*

- Methods: "not covered by a CT scan" -- suggest an alternative verb to covered

*Segmentation of the labels for non-brain anatomical regions of interest was performed using 3D Slicer (46). Two additional tools were used to segment the upper skull that was not CT scanned (51,52).*

- Methods: "P.E.G, and M.H.L." to "P.E.G. and M.H.L." (note this typo occurs twice in this section)

*The resulting values were compared with labels already adjusted and confirmed by two sub-specialty certified neuro-radiologists, P.E.G. and M.H.L. through an iterative process, which provided the most accurate information of the MRI and CT segmentations without a bias. The following DSC definition was used:*

*where X is the manually labeled region by one of our trained segmentors, Y is the region of interest volume that was confirmed as a ground truth by certified neuro-radiologists, P.E.G. and M.H.L., also through an interactive process (21). DSC ranges from a value of 0 to 1, greater than 0.8 was considered as acceptable, whereas 0.9 was considered excellent.*

- Methods: there is no need to repeat the phrase "two experienced, sub-specialty certified neuro-radiologists" or similar phrases many times. After first stating this, the authors can simply state "certified neuro-radiologists" or simply "neuro-radiologists". Variations of this lengthy phrase also appear later.

*‘The resulting values were compared with labels already adjusted and confirmed by certified neuro-radiologists, P.E.G. and M.H.L. through an iterative process, which provided the most accurate information of the MRI and CT segmentations without a bias.’*

*where X is the manually labeled region by one of our trained segmentors, Y is the region of interest volume that was confirmed as a ground truth by certified neuro-radiologists, P.E.G. and M.H.L., also through an interactive process (21).*

*Segmented tissues were then adjusted and confirmed independently by certified neuro-radiologists, P.E.G. and M.H.L, also through an iterative process, following a “pass or fail” method. When both raters scored each tissue with “pass”, the segmentation was finalized*

*The inter-operator variability indices (****Fig 7)*** *were calculated for each segmentor independently and compared against the ground truth that was adjusted and confirmed by certified neuro-radiologists through an iterative process (21).*

- Methods: "Sim4life" to "Sim4Life"

*‘A Bloch simulation tool kit in Sim4Life (66) called SYSSIM was used in order to generate a synthetic MRI image for additional validation.’*

- Methods: "extracted, and curvature smoothing was done" to "extracted, curvature smoothing was done"

*‘Once a surface was extracted, curvature smoothing was applied using a constrained Laplacian surface smoothing method with small tolerance using geometric flow that prevents volume shrinking (75).’*

- Regarding the above suggestion, the verb "done" is used many times throughout the manuscript. More precise verbs would improve the readability.

*‘Once a surface was extracted, curvature smoothing was applied using a constrained Laplacian surface smoothing method with small tolerance using geometric flow that prevents volume shrinking (75).’*

- Methods: "Technical Specification (ISO/TS) 10974 Technical Specification" seems to have unnecessary repeated words

*‘Examples of simulations followed the newest (2018) International Organization for Standardization Technical Specification (ISO/TS) 10974 – assessment of the safety of magnetic resonance imaging for patients with an active implantable medical device (83).’*

- Methods: Mentioning Sim4Life as a tool used in the study does not require a reference to the product [65] unless the authors are referencing a specific detail in the documentation.

*Thank you for this suggestion. The citation of the Sim4Life program is one of the requirements to maintain the ‘Sim4Life Science Program’. We cited once at the beginning of the manuscript and removed the additional citation of Sim4Life.*

- Results: "result was put together" -- suggest rewording

*Brain CSF was initially segmented based on the IR sequence of the head. The initial CSF segmentation was combined and reevaluated with the segmentation of the neural brain structures and the vessels of the brain using the MPRAGE sequence.*

- Results: "4.2 % and 3.3 larger" to "4.2% and 3.3% larger"

*The same process was followed for the kidneys for which the left and the right kidney weighed 48 g and 48.6 g, respectively, being 4.2 % and 3.3 % larger than the literature values. Adrenal glands were segmented after the segmentation of the kidneys, and their combined weight was 6.19 g, which is only 0.01% different for the reference values (89).*

- Results: "of they the thymus" to "of the thymus"

*IR of the chest was first used for the segmentation of the thymus, and the tissue was finalized using the whole-body T1, with its weight being 19.8 g, which is only 0.1% smaller than the reference value.*

- Results: "were found high in the bone" to "were found in the bone"

*The highest permittivity ratios (i.e., adult rat vs. ten days old rat) were found in the bone, the brain, and in the skin as 1.99, 1.33, and 1.29, respectively, compared to the averaged soft tissue ratio of 1.22. The conductivity ratios of 2.4, 1.6, and 1.5 were calculated for the bone, the brain, and the skin, respectively.*

- Results: "of a MARTIN of a pilot use case of with and without" to "of a pilot use case of MARTIN with and without"

*The head averaged SAR (SARhead), whole-body averaged SAR (SARwb), 10gSAR, and 10g averaged rms E-field (10gE) of a pilot use case of MARTIN with and without a VNS implant are reported in Table 7.*

- Discussion: "inter-subject data" should be "intra-subject data", since it was the same subject, at least that is how it reads now

*The partial-body CT scan was conducted 5-month before the MR scan that caused size difference within intra-subject data, and also the position of the subject, which made it difficult to register the entire CT images into MRI at once. CT was still valuable information to capture the boundary of calcified bone within the coverage of scan (e.g., hands and legs were missing) followed by the final manual refinement based on the T1 whole-body completed the registration.*

- Discussion: "opportunity for ones to separate" to "opportunity for one to separate"

*The automated segmentation process offers a reduced time of work and also provides an opportunity for one to separate anatomical regions with limited knowledge of anatomical atlases (e.g., separation of white matter and gray matter using T1 and T2 contrast in MRI).*

- Discussion: "other regions of and other limbs" -- remove "of"

*Multiple references were used for hand bones and other regions and other limbs (65,103,104) to increase the confidence in the segmentation of extremity bones in MR data (****S2 Fig 2****).*

- Discussion: "reported in this study with MARIN" -- typo for MARTIN

*SAR_head_, SAR_wb_, and 10gSAR in 1.5T reported in this study with MARTIN and Nina without an implant were consistent with that of the Baby model developed by Helmholtz Zentrum München (29) reported in 2015 by Malik et al. (111), which was a valuable reference to compare the simulation setup and use of dielectric properties conversion.*
